# How a Population Health Approach Improves Health and Reduces Disparities: The Case of Head Start

**DOI:** 10.5888/pcd13.150565

**Published:** 2016-01-21

**Authors:** Steven M. Teutsch, Ariella Herman, Carol B. Teutsch

**Affiliations:** Author Affiliations: Steven M. Teutsch, Fielding School of Public Health, University of California, Los Angeles, California, and Public Health Institute, Oakland, California; Ariella Herman, Carol B. Teutsch, Anderson School of Management, University of California, Los Angeles, California.

Good health is a life-long process. Many of the most critical behaviors are established in early childhood and need reinforcing at each life stage. To encourage physical activity, for example, young children should engage in fun, active play daily; schools should ensure that physical activity is a normal part of daily life; parents should participate in physical activity at work, home, or both and include their children; and elders should be encouraged to stretch, move, and improve their strength. Children who understand and demonstrate healthful behaviors will bring those messages home to parents. They can advocate for healthful food (and perhaps not pester parents for unhealthful foods!), encourage parents to stop smoking for their children’s sake, and seek fun active activities rather than more screen time. Thus, the family unit with young children can become an important force for sustaining more healthful living. Amplifying the positive impacts of parents as a child’s first teacher can set a child on a healthful trajectory for life.

The health of Americans sadly lags that of most other developed countries (1) and is beset by large disparities among racial/ethnic groups and the socially disadvantaged. Recognition that this problem cannot be solved by better and more accessible medical care alone has led to the resurgence of interest in population health (2) and its underlying determinants: behaviors and social and environmental conditions. The ecologic model of health recognizes the biological determinants of health and emphasizes the importance of family, community, institutions, laws, policies, and customs as well as the built and natural environments. Intrinsically it means that improving health and even disease outcomes requires intervention at multiple levels. According to the Guide to Community Preventive Services, effective interventions almost invariably require multiple components (3). To continue with the physical activity example, an effective strategy might include physician counseling, physical education programs in school, easy access to parks and recreation facilities, safe neighborhoods, complete streets (4), active transportation, education about the importance of physical activity, and encouraging families to participate in activities together. Implementing such multipronged interventions in the community can be challenging. Yet multicomponent and multigenerational approaches have the potential not only to improve the health of children but also can use parents’ and grandparents’ motivation to raise healthy children to improve their own health behaviors and thereby accelerate changes in social norms.

Health literacy, the ability of people to understand health and disease that empowers them to take action, is important, but by itself is insufficient. It can provide individuals and families with needed health information, motivate them, enhance communication with providers, and facilitate peer-to-peer communication. Although awareness is growing that health literacy is integral to health, it needs to be embedded in a more comprehensive set of policies, programs, and conditions that promote health and enable individuals to make healthful choices the easy choices.

Vulnerable low-income families in the United States have a disproportionate share of unhealthful determinants and poor health outcomes. While lack of access to medical care is one barrier, health literacy, access to culturally relevant programs, ability to satisfy basic needs, and financial insecurity are other typical barriers this population faces. Head Start and the Special Supplemental Nutrition Program for Women, Infants, and Children (WIC), along with many other agencies, foundations, and community-based organizations, provide health and education services to low-income families. Nurse Family Partnership provides public health nursing and health education services to low-income women. United Way provides workforce development, financial literacy, and health education across the country. First 5 California provides resources and tools to parents to help them navigate health and education for their children aged less than 5 years.

But Head Start is one of the longest-standing programs providing health and education services to low-income families. It serves approximately 1 million children aged 0 to 5 years and requires its grantees to coordinate health-related services such as basic screenings, health education, and referrals to health care providers as well as coordinating support for routine home visits and parent education workshops to understand the needs of families and children and promote preventive health services.

A 2001 survey of Head Start directors in the Johnson & Johnson Head Start Fellows program found that although grantees had access to health education materials and resources to conduct health education trainings, these sessions were often poorly attended and the materials were not well understood (5). In response, the University of California, Los Angeles/Johnson & Johnson Health Care Institute (HCI) was formed to rectify these limitations and improve health literacy among Head Start families.

HCI is now working in partnership with the American Academy of Pediatrics’ National Center on Early Childhood Health and Wellness. It uses the structured framework it developed for health promotion that builds staff leadership capacity and trains Head Start staff to implement health promotion programs for their families using culturally adapted, low-literacy materials on topics such as management of common childhood illnesses, effective use of the clinical care system, obesity prevention, home safety, mental health, and oral health. Family trainings capitalize on families’ innate motivation to care for their children. The trainings include experiential group learning activities, hands-on skill-building, and creation of social networks. This approach helps not only parents and children but also Head Start staff, who gain knowledge to care for their own children. The staff also see themselves as stronger role models for healthful living for their families, which also motivates family participation and engagement (6). The health literacy components are complemented by environmental changes, such as providing more healthful foods and more physical activity at schools, visiting community grocers to learn about how to buy and prepare healthful, affordable foods, and working with grocers to sell more healthful products (7). Since 2001, HCI trained staff from 300 grantees, who reached more than 120,000 vulnerable families across the United States.

Results of the health promotion program among more than 9,000 families at 55 sites were impressive: emergency department visits declined by 58% and school days missed were reduced by 29%, enhancing school readiness (8). Parents’ work-loss days decreased by 42% (8); such decreases are critical for low-wage workers at high risk of losing those jobs if they miss work, thus enhancing job security and incomes. Since income is itself a determinant of health and loss of a job a major life stressor, these indirect benefits of the program are also critical to a family’s well-being. Over the longer term, life-long improvements in health behaviors, higher graduation rates, and better job opportunities in healthier, more supportive communities should pay off in reduced rates of major chronic diseases as well. Partially in response to these successes, Head Start has incorporated health literacy into its proposed revised performance standards.

A comprehensive health literacy program coupled with population health interventions can reduce short-term and long-term disparities between the general population and Head Start children, families, and staff. Short-term benefits include fewer unnecessary emergency department visits, greater self-confidence for parents, better relationships and engagement with Head Start staff, and better health decision making. In the longer term, more healthful behaviors will contribute to reduced rates of cardiovascular disease and obesity. Because the social capital for implementing these programs resides in each Head Start agency, the programs are scalable and sustainable.

As this multidimensional health literacy and population health intervention illustrates, once parents have the knowledge, tools, and motivation to protect the health of their children, and barriers are removed, meaningful change can occur. When provided with simple health information in a low-literacy format, thermometers, and tips on how to improve health behaviors on a limited budget, along with changes in their school and community environments, parents were able to take positive action. Their empowerment and knowledge also better enables them to more appropriately access the clinical care system, ask appropriate questions, and understand and adhere to clinical recommendations. We can help set entire families and communities on a better health trajectory. A life-course approach and improved health literacy coupled with healthful programs and policies can reduce health disparities and make a real difference in people’s lives.
